# Involvement of Polyamines From Cardiac Mast Cells in Myocardial Remodeling Induced by Pressure Overload Through Mitochondrial Permeability Transition Pore Opening

**DOI:** 10.3389/fcvm.2022.850688

**Published:** 2022-04-11

**Authors:** Xiaolan Xiong, Junming Li, Shizhong Zhang, Xiaoli Jia, Chao Xiao

**Affiliations:** ^1^Third-Grade Pharmacological Laboratory on Traditional Chinese Medicine China Three Gorges University, Yichang, China; ^2^Medical College, China Three Gorges University, Yichang, China; ^3^The Second People’s Hospital of Yichang, Yichang, China; ^4^The First People’s Hospital of Yichang, Yichang, China

**Keywords:** pressure overload, cardiac remodeling, mast cell, polyamine, MPTP

## Abstract

**Objective:**

Polyamines mainly contain spermine (SPM), spermidine (SPD), and putrescine (PUT). Many research results suggest that polyamines participate in cell proliferation, differentiation, and the regulation of gene expression, and have a close relationship with the occurrence and development of many diseases. However, the role and possible mechanisms of action of polyamines from cardiac mast cells in myocardial remodeling induced by pressure overload remain to be elucidated.

**Methods:**

Pressure overload was induced by abdominal aortic constriction (AAC). Toluidine blue staining was used to visualize mast cells in cardiac tissue. The polyamine content of cardiac tissue was analyzed using high-performance liquid chromatography. Opening of the mitochondrial permeability transition pore (MPTP) was determined by the Ca^2+^-induced swelling of isolated cardiac mitochondria, measured as a reduction in A_520_.

**Results:**

Compared with sham rats, the cardiac mast cell density, the polyamine content (PUT, SPB, and SPM), and myocardial MPTP opening in rats with AAC were significantly increased (*P* < 0.05), and were accompanied by increased myocardial fibrosis and heart weight/body weight ratio. Intraperitoneal injection of polyamines mimicked these results, and these effects were reversed by cromolyn sodium, a mast cell stabilizer (*P* < 0.05). Myocardial MPTP opening increased in rats with AAC (*P* < 0.05), and the three polyamines also increased myocardial MPTP opening (*P* < 0.05).

**Conclusion:**

Mast cell-derived polyamines are involved in pressure overload-induced myocardial remodeling by increasing opening of the MPTP.

## Introduction

A large number of studies have shown that cardiac mast cells play an important role in cardiac remodeling (1). Experimental studies have found that inhibition of mast cell degranulation significantly reduces cardiac remodeling in several heart diseases including hypertension, ischemia/reperfusion injury, and transplantation/rejection disease (1–6). Studies in mast cell-deficient animals have shown that cardiac weight is significantly reduced after hypertension and ischemia-reperfusion (7). The process of mast cell involvement in cardiac remodeling is closely associated with the material released by mast cell degranulation after activation, which include a variety of active substances including histamine, cytokines, phospholipase, proteases, and fatty-acid metabolites (8–10). Although studies have suggested that some of these components such as proteases and cytokines activate collagenases and matrix metalloproteinases that are involved in cardiac remodeling (11, 12), the specific substances that induce remodeling after degranulation of cardiac mast cells still need further investigation. A recent study has established for the first time that, in addition to the above known substances, polyamines are also released by mast cells after activation (13).

Interestingly, in cases of cardiac ischemia and increased pressure overload, there is an increase in the polyamine content of heart tissue in addition to an increase in mast cell density (13–15). And many research results suggest that polyamines are involved in the cardiac remodeling process induced by stress (16, 17).

Polyamines mainly contain spermine (SPM), spermidine (SPD), and putrescine (PUT), which are small organic molecules widely found in eukaryotic cells. In the organism, ornithine is decarboxylated to form PUT under the action of ornithine decarboxylase, PUT produces SPD under the action of SPD synthase, and SPD produces SPM under the action of SPM synthase. Polyamines not only participate in many important physiological processes such as cell proliferation, differentiation, and the regulation of gene expression, but also have a close relationship with the occurrence and development of many diseases (18–20). It has been reported that the cardiac polyamine content is higher in spontaneously hypertensive rats, and the administration of SPM to such rats significantly increases their blood pressure (21). Studies on vascular polyamines in hypertensive rats have also shown similar changes in cardiac polyamines in rats with spontaneous hypertension, and this change is accompanied by changes in vascular function (22). These results suggest that polyamines are closely associated with hypertension, a stress-loading heart disease, and have important research value. Studies have shown that polyamines are involved in the cardiac hypertrophy induced by the stimulation of norepinephrine and its receptor system (23), suggesting that polyamines play an important role in cardiac remodeling. Research has shown that the respiratory amine concentration increases in tracheal cystic fibrosis (24), suggesting that polyamines participate in the process of tissue fibrosis. However, the exact role and mechanisms of action of polyamines in cardiac remodeling induced by pressure overload remain unclear.

Research on cardiac remodeling mechanisms indicates that the mitochondrial permeability transition pore (MPTP) is involved in the process of cardiac remodeling: in mice: with knockout of the MPTP opening regulatory protein SIRT3, the MPTP opens easily, and this is accompanied by cardiac hypertrophy and fibrosis (25). In mice with gene knockout of the MPTP regulatory protein cyclophilin D, stress overload results in more severe cardiac hypertrophy and fibrosis (26, 27). These findings suggest that abnormal opening of the MPTP leads to more severe cardiac hypertrophy and fibrosis. However, these experimental data were all obtained under a condition of artificially changing the opening of the MPTP. Whether the cardiac remodeling induced by pressure overload is accompanied by an abnormal change of the MPTP, and if so, what the underlying possible mechanism is, remain unclear.

Based on the above, we hypothesized that pressure overload induces cardiac mast cell aggregation in cardiac tissue and polyamines released from these cells may be involved in the cardiac remodeling induced by pressure overload through the regulation of MPTP opening. Here, we tested this hypothesis using an animal model of pressure overload induced by abdominal aortic stenosis and mitochondria isolated from the rat heart.

## Materials and Methods

### Animals

Male and female Sprague–Dawley rats (160–170 g) were obtained from the Animal Center of China Three Gorges University, housed under standard conditions, and maintained on commercial rat chow and tap water *ad libitum*. All experimental protocols were approved by the Institutional Animal Care and Use Committee of China Three Gorges University and adhered to the National Institutes of Health guidelines for the use of experimental animals.

### Pressure-Overload Rat Model

The rat model of pressure overload was induced by abdominal aortic constriction (AAC) (28). Briefly, rats were anesthetized with 10% chloral hydrate (350 mg/kg, i.p.), and the aorta was dissected above the two renal arteries. A silver clip (0.70 mm internal diameter) was placed on the abdominal aorta above the level of the left renal artery.

### Study Design

Rats were randomly divided into the following groups: the AAC group, in which the rats were subjected to AAC (half male and half female); the sham group, in which the rats underwent the same surgery as in the AAC group but without placement of the abdominal aortic clip; the CS group, in which the rats were treated with cromolyn sodium (CS, 25 mg/kg/day, i.p., half male and half female) (29) after AAC surgery; and the polyamine groups, in which the rats were treated with polyamines (PUT, SPD, or SPM, 10 mg/kg/day, i.p., half male and half female) (30–32). All hearts were then carefully removed after 4 or 8 weeks of treatment. The middle section of the left ventricle was immediately divided into 2-mm thick transverse sections and immersed in 10% neutral buffered paraformaldehyde for histological analysis. Other tissue samples were flash-frozen in liquid nitrogen and stored at -80°C until Assays were performed.

### Chemicals

Putrescine, SPD, SPM, and 1,6 hexamethylene diamine were from Sigma Chemical Co.; CS, chymase, and histamine were from Macklin; Masson’s trichromatic staining solution and toluidine blue color solution were from Google Biology; and hematoxylin-eosin staining solution was prepared in the laboratory.

### Measurement of Rat Myocardium Polyamine Levels Using High-Performance Liquid Chromatography

After euthanasia, hearts were excised and weighed. In each group, ∼150 mg of the left ventricle was taken, and cold perchloric acid (0.3 mol/L) was added. Then a tissue homogenate was prepared in an ice water bath, and centrifuged before collecting the supernatant. The perchloric supernatant was alkalinized by 2 mol/L NaOH, followed by the addition of benzoyl chloride to form derivatives. The derivatives were separated and determined on a Waters 2,690 reverse-phase high-performance liquid chromatography system equipped with an auto-sampler, column oven, ultraviolet (UV) detector and a reversed-phase Luna C18, analytical column (150 × 4.6 mm id, 5 μm particle size). Data and spectra were recorded and analyzed by the Millennium 32 Chromatography Workstation (Waters, United States). Chromatographic separation was achieved with isocratic elution using a mobile phase composed of water (60%) and acetonitrile (40%) at 1.0 ml/min.

### Histological Analysis

Transverse sections at the mid-ventricular level (2 mm) were fixed in 10% neutral buffer. Then, histological analyses were done using standard protocols. Masson’s trichrome staining (33) was used to assess the area of fibrotic infiltration in the wall of the left ventricle. The collagen fibers stained blue, in contrast to red-stained myocardium. In each specimen, the degree of fibrosis was quantified independently by two pathologists in a blinded fashion. Each section was imaged using an Olympus BX53 camera (Tokyo, Japan). The area of myocardial fibrosis was quantified using Image-Pro Plus 6.0 (Media Cybernetics, Rockville, MD, United States). The area fraction of myocardial fibrosis was expressed by the fractional area (percentage of total surface area) of the section occupied by collagen deposition. The mast cells in the myocardium were stained with toluidine blue in paraffin sections. Mast cells were counted in a high-power field of view (400× magnification). Mast cell density was quantified by counting the number of toluidine blue-positive mast cells in five consecutive fields.

### Mitochondria Preparation

Mitochondria were isolated from the hearts of all groups with the same protocol as before (34). In brief, heart tissue was homogenized in ice-cold buffer containing 160 mmol/L KCl, 10 mmol/L EGTA (pH 7.4), and 0.5% fatty-acid-free bovine serum albumen. The homogenate was centrifuged at 1,000 *g* for 10 min at 2°C, and the supernatant was centrifuged at 8,000 *g* for 10 min at 2°C. The pellet was re-suspended in buffer containing 320 mmol/L sucrose and 10 mmol/L Tris–HCl (pH 7.4), and centrifuged at 8,000 *g* for 10 min at 2°C. Then we acquired the purified mitochondria. The protein concentration in samples was determined using the Lowry method. Each sample used 150 μl of protein solution (i.e., mitochondria). All experiments were repeated at least five times and done in triplicate.

### Mitochondrial Permeability Transition Pore Opening Assay

Mitochondrial permeability transition pore (MPTP) opening was detected by assessing mitochondrial swelling, which was determined by a decrease in light absorbance at 520 nm (A_520_). A UV-2100 UV-Vis spectrophotometer (Unocal Instruments) was used to measure OD changes at 520 nm for 15 min. To induce mitochondrial swelling, CaCl_2_ (200 μM) was applied to microplates containing mitochondria. The effects of PUT (10 μM), SPD (10 μM), and SPM (10 μM) on MPTP opening were recorded.

### Statistical Analysis

All values are expressed as the mean ± SD. Statistical comparisons were performed by one-way analysis of variance and followed by the Newman–Keuls test. Differences were considered significant when *P* < 0.05.

## Results

### Pressure Overload Increases Mast Cell Density in Cardiac Tissue

**FIGURE 1:**
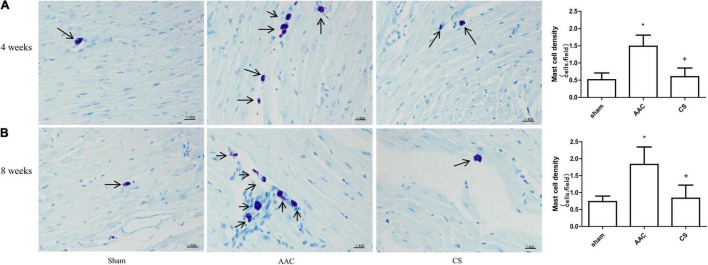
Mast cell density changes in cardiac tissue. **(A,B)** Mast cell density 4 weeks **(A)** and 8 weeks **(B)** after abdominal aortic constriction; mast cells indicated by arrows stained dark purple with toluidine blue (400× magnification). AAC, abdominal aortic constriction; CS, cromolyn sodium; *n* = 7 per group. *P < 0.05 *vs* sham group; ^+^*P* < 0.05 *vs* AAC group; Scale bars, 100 μm.

Compared with sham rats, the cardiac mast cell density was significantly increased in rats 4 or 8 weeks after AAC surgery, and this effect was reversed by treatment with CS, a mast cell stabilizer (Figure 1).

### The Increased Polyamine Content in Cardiac Tissue Induced by Abdominal Aortic Constriction Is Attenuated by Inhibition of Mast Cells

**FIGURE 2:**
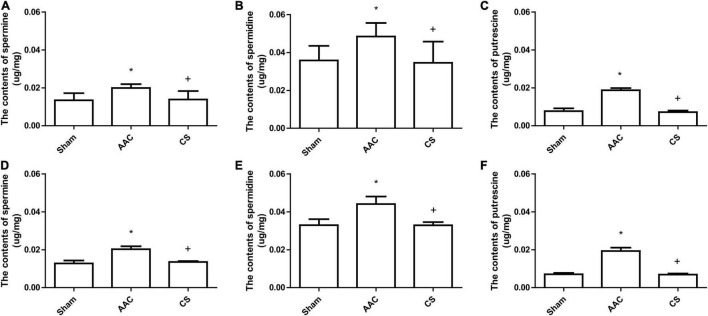
Polyamine content in cardiac tissue from rats 4 weeks **(A–C)** or 8 weeks **(D–F)** after AAC surgery. AAC, abdominal aortic constriction; CS, cromolyn sodium; *P < 0.05 *vs* sham group; ^+^*P* < 0.05 *vs* AAC group. *n* = 9 per group.

Compared with sham rats, 4 or 8 weeks after AAC surgery, the polyamine content of cardiac tissue increased significantly, and this increase was attenuated by inhibition of mast cells with its stabilizer, indicating that the increase of polyamines is dependent on mast cell activation (Figure 2).

### Exogenous Administration of Polyamines Mimics the Cardiac Remodeling Induced by Pressure Overload

**FIGURE 3:**
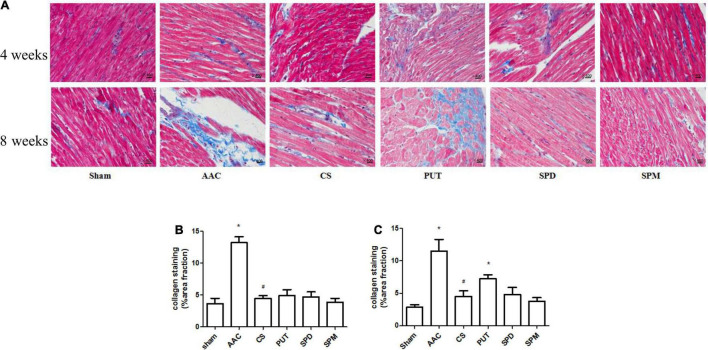
Collagen deposition in cardiac tissue. **(A)** Representative images of collagen staining (400× magnification). Heart sections stained with Masson’s trichrome; collagen is stained blue. AAC, abdominal aortic constriction; CS, cromolyn sodium; PUT, putrescine; SPM, spermine, SPD, spermidine. **(B,C)** Statistical analysis of collagen deposition after 4 weeks **(B)** and 8 weeks **(C)** of treatment with polyamines. *P < 0.05 *vs* sham group; ^#^*P* < 0.05 *vs* AAC group. *n* = 8 per group. Scale bars, 100 μm.

Compared with sham rats, the heart-weight to body-weight (HW/BW) ratio in rats with AAC increased significantly, and this was attenuated by administration of a mast cell stabilizer (Table 1). However, exogenous administration of polyamines for 4 or 8 weeks not only caused an increase of the HW/BW ratio similar to that in AAC rats, but also showed a significant increase of polyamine content in cardiac tissue (Figure 3).

**TABLE 1:** Heart-weight to body-weight (HW/BW) ratio.

Parameters	Sham group	AAC group	CS group	PUT group	SPD group	SPM group
**4 weeks HW/BW**	2.90 ± 0.16 *n* = 12	3.49 ± 0.2* *n* = 21	3.16 ± 0.20^+^ *n* = 23	3.48 ± 0.40* *n* = 18	3.15 ± 0.44* *n* = 18	3.07 ± 0.29* *n* = 18
**8 weeks HW/BW**	2.69 ± 0.22 *n* = 15	3.56 ± 0.35^#^ *n* = 14	2.92 ± 0.26^∧^ *n* = 14	3.25 ± 0.25^#^ *n* = 14	3.07 ± 0.27^#^ *n* = 15	3.19 ± 0.47^#^ *n* = 10

AAC, abdominal aortic constriction; CS, cromolyn sodium; PUT, putrescine; SPM, spermine, SPD, spermidine. Data are expressed as the mean ± SD. ^*,#^P < 0.05 vs Sham group; ^+,∧^P < 0.05 vs AAC group.

Collagen deposition, a marker of interstitial fibrosis, was also significantly enhanced in cardiac tissue in AAC rats in comparison with that in sham rats, and this increase was attenuated by the mast cell stabilizer. Exogenous administration of PUT caused an increase of collagen deposition in cardiac tissue similar to that in AAC rats, but SPM and SPD did not (Figure 3).

### Polyamines From Mast Cells Increase Cardiac Mitochondrial Permeability Transition Pore Opening

**FIGURE 4:**
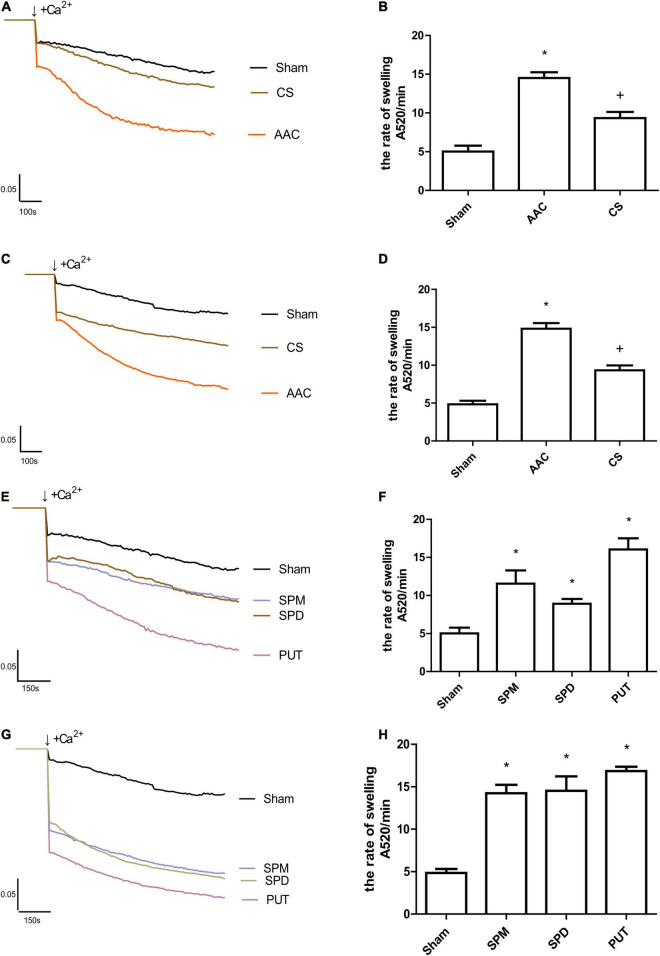
Changes in Ca^2+^-induced mitochondrial swelling rate. **(A,B)** Tracings of cardiac mitochondrial swelling **(A)**, and statistics of swelling rates **(B)** 4 weeks after different treatments; **(C,D)** Tracings **(C)** and statistics **(D)** of cardiac mitochondrial swelling 8 weeks after different treatments; **(E,F)** Tracings **(E)** and statistics **(F)** of cardiac mitochondrial swelling 4 weeks after administration of polyamines; **(G,H)** Tracings **(G)** and statistics **(H)** of cardiac mitochondrial swelling 8 weeks after administration of polyamines. AAC, abdominal aortic constriction; CS, cromolyn sodium; PUT, putrescine; SPM, spermine; SPD, spermidine; *n* = 7 per group.; *P < 0.05 *vs* Sham group; ^+^*P* < 0.05 *vs* AAC group.

To assess the effects of polyamines on MPTP opening, we used the Ca^2+^-induced mitochondrial swelling method, and the results showed that opening of the MPTP was significantly increased in cardiac mitochondria from rats 4 or 8 weeks after AAC; however, this phenomenon was absent in cardiac mitochondria from rats treated with CS, the mast cell stabilizer (Figures 4A–D). To determine the role of polyamines in this process, we further assessed the effects of exogenous administration of polyamines on MPTP opening, and the results showed that the MPTP opening after continuous administration of PUT, SPM, or SPD was also significantly increased compared with sham rats (Figures 4E,F). Among the three polyamines, PUT made the greatest contribution to MPTP opening (Figures 4G,H).

## Discussion

Our present results show that pressure overload can induce the recruitment of mast cells in cardiac tissue and the release of polyamines, which then participate in the process of myocardial remodeling by regulating opening of the MPTP.

In the present study, the recruitment of mast cells in cardiac tissue was demonstrated by the increased cardiac mast cell density 4 or 8 weeks after AAC in rats, and this was accompanied by increases in the HW/BW ratio and cardiac fibrosis. These results are similar to those in other reports (28, 35, 36). Interestingly, in our study, these effects were attenuated by inhibition of mast cell activation, suggesting that the activation and degranulation of mast cells are involved in the cardiac remodeling induced by pressure overload.

Although the activation of mast cells releases a variety of components including histamine, cytokines, phospholipase, proteases, and fatty-acid metabolites (37), we found an increase of polyamines in cardiac tissue after pressure overload. To confirm that these polyamines were released from mast cells, we inhibited mast cells using a stabilizer, and the results showed that the increase of polyamines was significantly attenuated, indicating that the increased polyamine content was from cardiac mast cells.

Research data has shown that the myocardial polyamine content is increased in hypertensive patients (21, 22), and that polyamines might be involved in the cardiac hypertrophy induced by norepinephrine and its receptor system (23), as well as being involved in fibrosis (24). To further investigate whether the increased polyamines participate in the cardiac remodeling induced by pressure overload, we assessed the effects of exogenous polyamines on heart remodeling, and the results showed that after 4 weeks of continuous administration of PUT, SPM, or SPD, not only the polyamine content in cardiac tissue but also the HW/BW ratio increased significantly, and after 8 weeks of continuous administration of PUT, myocardial fibrosis and the number of myocardial mast cells increased as much as that in rats with pressure overload. In order to further investigate whether inhibiting the release of polyamines from mast cells can decrease the myocardial fibrosis, we inhibited degranulation of mast cells by using mast cell stabilizers. The results showed a great decrease of polyamines, which was associated with a decrease of myocardial fibrosis. All the above experimental results indicated that polyamines from mast cells are involved in the cardiac remodeling induced by pressure overload.

Studies have shown that MPTP opening is involved in the process of cardiac remodeling: in mice with knockout of an MPTP opening regulatory protein, the MPTP opens more easily, and this is accompanied by cardiac hypertrophy and fibrosis (25). In mice with gene knockout of cyclophilin D, an MPTP regulatory protein, more severe stress overload-induced cardiac hypertrophy and increases in fibrosis also occur (26, 27). These findings suggested that abnormal opening of the MPTP is closely associated with cardiac hypertrophy and fibrosis. However, these experimental data were all collected under conditions in which the MPTP opening was artificially altered. In the situation of pressure overload, whether the cardiac MPTP is in an abnormal open state, and particularly whether cardiac mast cells participate in the regulation of MPTP opening is still unclear. We found that opening of the cardiac MPTP in AAC rats increased significantly, and inhibition of mast cells attenuated the MPTP opening, suggesting that mast cell recruitment and activation can induce opening of the MPTP. Since we detected a significant increase in the polyamines released from cardiac mast cells after pressure overload, we further investigated whether polyamines released by mast cells participate in regulating the MPTP. We did experiments to measure the effects of PUT, SPD, and SPM on MPTP opening in mitochondria from normal rats, and the results showed that these polyamines significantly enhanced opening of the MPTP, indicating that polyamines from mast cells mediate myocardial remodeling through increasing abnormal opening of the MPTP. Although we did not further investigate the possible underlying mechanisms of polyamine-induced MPTP opening, according to the published reports, the corresponding underlying mechanisms may be complex: endoplasmic reticulum stress, oxidative stress, intracellular Ca^2+^ homeostasis, and even autophagy may all participate in the regulation of MPTP opening (38–45). Thus the exact mechanisms need to be further examined.

Activation of mast cells also releases other substances, mainly 5-HT, chymase, and histamine, and whether these substances participate in the abnormal opening of the cardiac MPTP need to be further examined.

Although we did not perform cardiac function tests, in another study on myocardial remodeling (the results have not yet been published), our group examined the cardiac function of rats with AAC, and found that 8 weeks after surgery, the cardiac function did not decline, although fibrosis had occurred.

In summary, although we cannot exclude the possibility that other substances from mast cells also participate in the regulation of cardiac MPTP opening, our results do suggest that polyamines derived from cardiac mast cells are involved in the adverse ventricular remodeling induced by pressure overload *via* increasing cardiac MPTP opening.

## Data Availability Statement

The original contributions presented in the study are included in the article/supplementary material, further inquiries can be directed to the corresponding author.

## Ethics Statement

The animal study was reviewed and approved by the Institutional Animal Care and Use Committee of China Three Gorges University.

## Author Contributions

XX, XJ, and CX collected and analyzed the data. JL helped to do some measurements. XX and SZ wrote the manuscript. All authors contributed to the article and approved the submitted version.

## Conflict of Interest

The authors declare that the research was conducted in the absence of any commercial or financial relationships that could be construed as a potential conflict of interest.

## Publisher’s Note

All claims expressed in this article are solely those of the authors and do not necessarily represent those of their affiliated organizations, or those of the publisher, the editors and the reviewers. Any product that may be evaluated in this article, or claim that may be made by its manufacturer, is not guaranteed or endorsed by the publisher.
